# Covariation of taxonomic and functional facets of β-diversity in Chilean freshwater fish assemblages: Implications for current and future processes of biotic homogenization

**DOI:** 10.1371/journal.pone.0281483

**Published:** 2023-02-09

**Authors:** Sergio A. Castro, Pablo Rojas, Irma Vila, Fabian M. Jaksic

**Affiliations:** 1 Laboratorio de Ecología y Biodiversidad, Universidad de Santiago de Chile, Santiago, Chile; 2 Departamento de Ciencias Ecológicas, Universidad de Chile, Santiago, Chile; 3 Center of Applied Ecology and Sustainability (CAPES), Pontificia Universidad Católica de Chile, Santiago, Chile; Universidade Regional Integrada do Alto Uruguai e das Missoes, BRAZIL

## Abstract

The biodiversity of assemblages that experience the introduction and extinction of species may lead to responses in two important facets: The taxonomic and functional diversity. The way in which these facets are associated may reveal important implications and consequences for the conservation of those assemblages. Considering the critical situation of freshwater fishes in continental Chile (30° – 56° S), we analyzed how the taxonomic (*TD*_β_) and functional (*FD*_β_) facets of β-diversity, and their components of turnover and nestedness, are associated. We evaluated changes in β-diversity (Δ*TD*_β_ and Δ*FD*_β_), turnover (Δ*TD*_*tur*_ and Δ*FD*_*tur*_), and nestedness (Δ*TD*_*nes*_ and Δ*FD*_*nes*_) in 20 fish assemblages from their historical (pre-European) to current composition. We also simulated future trends of these changes, assuming that native species with conservation issues would become extinct. Our results show that the fish assemblages studied are in a process of loss of β-diversity, both in taxonomic and functional facets (Δ*TD*_β_ = -3.9%; Δ*FD*_β_ = -30.4%); also, that these facets are positively correlated in the assemblages studied (*r* = 0.617; *P* < 0.05). Both components showed by loss in nestedness (Δ*TD*_*nes*_ = -36.9%; Δ*FD*_*nes*_ = -60.9%) but gain in turnover (Δ*TD*_*tur*_ = 9.2%; Δ*FD*_*tur*_ = 12.3%). The functional β-diversity decreased more than the taxonomic (Δ*FD*_β_ > Δ*TD*_β_), which was caused chiefly by six exotic species of Salmonidae, whose geographical spread was wider and that at the same time shared several morpho-functional traits. Our forecasts, assuming an intensification in the extinction of Endangered and Vulnerable native species, indicate that the process of homogenization will continue, though at a lower rate. Our study shows that the freshwater ichthyofauna of continental Chile is undergoing biotic homogenization, and that this process involves the facets of taxonomic and functional β-diversity, which are show high correlation between historical and current compositions. Both facets show that process is influenced by nestedness, and while turnover contributes to differentiation (both taxonomic and functional), its importance is overshadowed by nestedness.

## Introduction

The spatial reorganization of global biodiversity is one of the main processes that define the epoch that we currently live in, the Anthropocene [1, 2]. The situation of freshwater fish assemblages is particularly worrying: They have high species richness all around the world [3] and are especially vulnerable to localized anthropic impact [4, 5]. Hundreds of fish species have been introduced to freshwater ecosystems in regions where their arrival would have been unlikely without human assistance [6], while others–usually native species with restricted distribution–have been eradicated or have become extinct [7, 8]. The introduction of exotic species and the disappearance of local taxa are causing the loss of β-diversity, in a process known as biotic homogenization [9, 10]. Because changes in beta-diversity imply important modifications in the structure and functioning of communities and ecosystems [11, 12], much interest has been focused on studying how these changes are expressed at the taxonomic, functional, and phylogenetic levels [13, 14].

Considering that “all species are not equal” (*sensu* White *et al*. [15]), each one should be viewed as possessing a unique set of functional traits [16] and its own evolutionary history [17]. Therefore, the addition or extinction of a given taxon should also entail a gain or loss in functional and phylogenetic diversity [11, 18], which are two integral facets of biodiversity. Currently, vigorous research is being carried out to understand how the phylogenetic (or taxonomic) dimension of β-diversity loss is associated with by changes in other aspects of biodiversity [19]. Among them, functional diversity has captured special interest, because it considers the traits associated with how a given species interacts with its environment, as well as those associated with the function, stability, and organization of the assemblage it belongs to [20–22]. For example, when an exotic species that possesses novel functional traits is introduced into two or more assemblages, its impact on functional diversity will likely be greater than on taxonomic diversity [23]. On the contrary, in assemblages where the different organisms face harsh environmental barriers or filters, only a few functional groups will be able to persist, leading to functional convergence that may in turn enable higher taxonomic diversity [24].

The measurements of β-diversity and its components of turnover and nestedness are especially informative indicators of the processes that determine the spatial organization of diversity [25–27]. While turnover describes the effective replacement of species/traits within assemblages–as a result of environmental filters, ecological interactions, and/or historical events–, nestedness provides information on changes in species/trait richness due to extinction/colonization events associated with limitations to dispersal [27, 28]. To date, relatively few studies have been conducted to analyze the covariation of taxonomic and functional β-diversity in terms of turnover and nestedness [29, 30]. Such two-pronged characterization is relevant for understanding the processes that determine and maintain β-diversity, as well as for implementing effective conservation measures [31]. Therefore, to establish the direction and magnitude of the taxonomic and functional changes that occur across space in freshwater fish assemblages, and the interdependence of these changes on the components of turnover and nestedness, are two important aspects to elucidate.

The freshwater fish assemblages of continental Chile represent an attractive biogeographical model within the South American Neotropics [32]. Because this country extends along a wide latitudinal gradient (18°-56° S), its hydrographic basins (and the fish assemblages that inhabit them) are spread out linearly from north to south, exposed to different geological settings, climatic regimes, and degrees of anthropic impact [33]. These assemblages, which contain 42 native species at the national level [34], exhibit a high level of endemism (77%; Ministerio del Medio Ambiente [35]) that is the result of their prolonged biogeographic isolation from the rest of South America [32, 36, 37]. Notwithstanding, the current state of conservation of the majority of these species is critical, with around 88% of them presenting conservation issues [38]. Additionally, the presence of 28 naturalized exotic fish species has aggravated the threat to the native ichthyofauna by competition and predation [38, 39]. Recent evidence indicates that the freshwater ichthyofauna of continental Chile has suffered one of the most severe cases of anthropic impact in the entire Neotropics [40, 41] and that its composition is undergoing both taxonomic [42] and functional homogenization [43]. Given the current situation, it becomes relevant to ask how taxonomic and functional diversity change in relation to each other along the wide geographic gradient of continental Chile. Additionally, given that the events of extinction/eradication (and not of new introductions of exotic species) represent the most likely compositional responses [39], it seems necessary to investigate the changes that are to be expected as a result of the disappearance of species that are currently experiencing conservation issues [19, 44].

In this study, we analyze changes in β-diversity of 20 freshwater fish assemblages in continental Chile. Specifically, we examine the covariation (if any) of the taxonomic and functional facets of β-diversity through the components of turnover and nestedness. We assess these relations in a temporal context, including the historical (*i*.*e*., prior to European colonization) and current fish assemblage composition (*i*.*e*., post-colonization), as well as two future scenarios that assume the intensification of extinction of native taxa. Based on data from other assemblages that have suffered extensive anthropic impact, β-diversity of freshwater fish in continental Chile is expected to be affected mostly by nestedness, and to a lesser extent by turnover [45–47]. Taking this comprehensive approach, we hope that our findings will help identify effective conservation measures to protect this unique ichthyofauna.

## Methodology

### The assemblages

**Fig 1:**
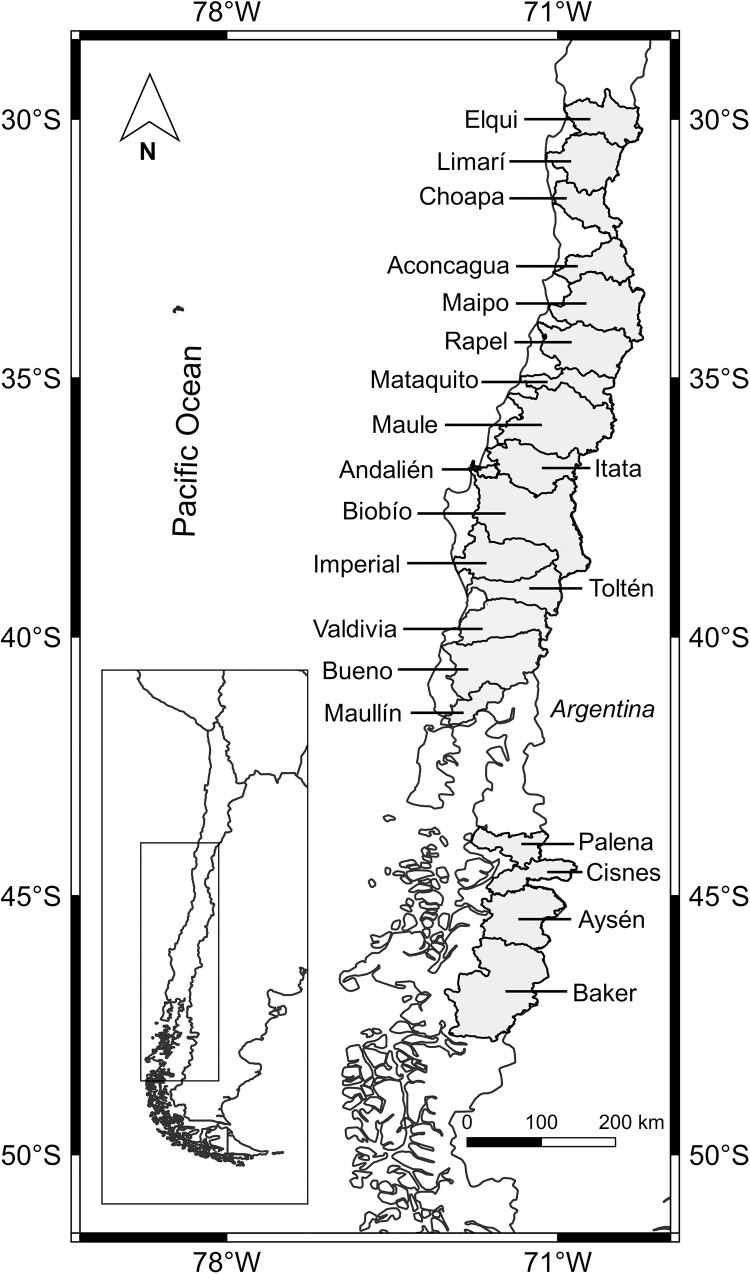
Location of the 20 basins studied in continental Chile. This map was created using information freely available on QGis and the public domain ‘Infraestructura de Datos Geoespeciales de Chile’ (https://www.ide.cl) and used under a CC-BY-4.0 licence.

We examined the composition of the fish assemblages of 20 hydrographic basins in continental Chile (Fig 1). Based on their surface area, these represent the main hydrographic basins in the country, extending over 225,705 km^2^, which corresponds to approximately 30% of the total surface area of continental Chile. These basins are located between latitudes 30° and 48°S (Fig 1) and all of them originate in the Andes Mountain Range, flowing east to west into the Pacific Ocean.

### Fish distribution and compositional scenarios

The freshwater fish composition of these basins was determined using our own database, supplemented with information found in the scientific literature and in environmental impact reports (see Castro *et al*. [42] for methodological details). In this study, we only considered fully freshwater species, excluding marine fish that are occasionally found in this environment. We considered as native the taxa that were present at the time of the European (Spanish) colonization of Chile in the 16^th^ century, and as exotic those that were introduced later and have naturalized populations in the basins (for details, see Castro *et al*. [42]). We assumed that the pre-European distribution of native fish was similar to that recorded at the beginning of the 20th century [42]. In Chile, this assumption is well supported because there is no evidence for extinction or translocation of native fishes during pre-European times [38–40, 43]. Thus, we tallied a total of 58 fish species, 30 of which are native and 28 are exotic (S1 Table).

The composition of the assemblages was set in two ‘species × basin’ distributional matrices. One of these was the historical matrix containing the most likely species composition of each assemblage prior to European colonization. According to our data, the historical α-diversity of the assemblages (*i*.*e*., the number of native species per basin) has remained unchanged to this day [45], with no cases of species translocations and a single case of extinction, that of *Diplomystes chilensis* [42, 48]. The current matrix contained the extant compositions and pools together the exotic and native species in each basin (excluding the extinct *D*. *chilensis*). In both historical and current compositional matrixes, we recorded the presence/absence of each species in the assemblages with a value of 1 (one) or 0 (zero), respectively.

We also analyzed the simulated effects of extinctions/eradications on the taxonomic and functional dimensions of β-diversity in two additional matrices: future-1 and future-2. In future-1 matrix, we simulated the extinction of the species with current Endangered conservation status (14 species; S1 Table), and in future-2 matrix both Endangered and Vulnerable species were simulated to become extinct (14 + 11 species; S1 Table). Following Matsuzaki *et al*. [49], these two scenarios assume the intensification of extinction/eradication processes based on the threat level associated with the current conservation status of each species. The latter was obtained for each species from the database of the Chilean Ministry of the Environment [35], a governmental department that has established this conservation classification at the basin level. Given that the introduction of new species into the country and the expansion of exotic taxa that are already present are under control thanks to current regulations [50, 51], we considered the exotic species composition not changing in the two future scenarios.

### Taxonomic and functional β-diversity

For quantifying β-diversity in its taxonomic and functional facets, we followed the proposal of Baselga [25, 28]. Although this approach has received some criticism, especially regarding the quantification of nestedness [52–55], its application has been widespread over the last decade (see Soininen *et al*. [56]; Wayman *et al*. [47]) in comparison to equivalent indexes (e.g., Sorensen’s). Thus, the use of the Baselga’s [28] approach favors comparative studies [25], but noting that its nestedness component is not a measure of true nestedness, but a measure of the fraction of the total dissimilarity that is caused by nestedness [53]. Nevertheless, for simplicity here we use the term nestedness to describe this nestedness resultant dissimilarity [44]. The β-diversity indicators, both taxonomic and functional, were obtained from those proposed by Baselga [57, 58] and Villéger *et al*. [59], respectively. These indicators are based on Jaccard’s index [22, 57], and so they belong to the same family (*sensu* [25]), thus allowing comparative analysis and exploration of their trends [60].

In order to evaluate taxonomic β-diversity (*TD*), we used the Jaccard index (*TD*_β_ = (*b + c*) (*a* + *b* + *c*)^-1^; [28]), with additive partition to calculate turnover (*TD*_*tur*_ = (2 min{*b*,*c*}) (a + 2 min{*b*,*c*})^-1^) and nestedness (*TD*_*nes*_ = (|*b*–*c*|) (*a* + *b* + *c*)^-1^) (*a*) (*a* + 2 min{*b*,*c*})^-1^) [28]. In these calculations, *b* and *c* represent the number of unique species for each pair of basins (*i*.*e*., excluding shared species), while *a* corresponds to the number of shared species between each pair of basins. We used betapart in R to calculate these indices [58]. Because their values range from 0 to 1, the difference (Δ) between them—calculated for the different temporal stages—provides information on the increase, persistence, or decrease in the taxonomic facet of β-diversity. Therefore, we calculated the differences between the values of the three β-diversity indexes to quantify their trends from the historical to the current, and to the future-1 and future-2 scenarios.

To determine the functional component of β-diversity, we created a species × traits matrix (S2 and S3 Tables). In it, the 58 recognized species were codified based on five traits associated with their life history [59]. These traits and their character states were (S2 Table): a) adult body length (standard maximum length, cm), b) migration type (1: non-migratory; 2: potamodromous; 3: diadromous-anadromous; 4: amphidromous-diadromous; 5: catadromous), c) adult diet (1: invertivorous; 2: invertivorous-herbivorous; 3: invertivorous-herbivorous-piscivorous; 4: invertivorous and occasionally piscivorous; 5: generalized carnivorous; *i*.*e*., feeds on invertebrates, fish, amphibians and/or mammals), d) adult use of the water column (1: demersal; 2: pelagic), and e) fecundity (1: < 500 eggs; 2: 500–1,000; 3: 1,000–2,000; 4: 2,000–10,000; all per reproductive event).

Character state information for each species was obtained from Habit *et al*. [36, 61], Link and Habit [62], Vila *et al*. [63, 64], and FishBase (www.fishbase.org; see [65]) (S3 Table). Except for body length, all characters showed ordinal variation. Once the character states were established for each species, we used Gower distance [66] to create a morpho-functional distance matrix between each pair of species and then calculated the volume of the convex surface of each assemblage based on the first three principal axes in a Principal Coordinates Analysis (PCoA) [67].

The calculations of functional richness using the quality.fspaces function of the mFD package in R 4.2.1 [68], allowed to calculate the optimal PCoA values to describe the morpho-functional space [69]. We calculated the turnover and nestedness components of the functional β-diversity using the beta.fd.multidim function [68]. We used the following algorithm to calculate functional β-diversity (*FD*_β_): *FD*_β_ = [(V(A_1_) + V(A_2_))– 2 V(A_1_ ∩ A_2_)] [(V(A_1_) + V(A_2_))–V(A_1_ ∩ A_2_)]^-1^ [65], where V(A_1_) and V(A_2_) represent the multidimensional volume of assemblages A_1_ and A_2_, and V(A_1_ ∩ A_2_) represents their shared multidimensional volume [65]. We partitioned *FD*_β_ into its components of turnover (*FD*_*tur*_ = [2 min(V(A_1_), V(A_2_))– 2 V(A_1_ ∩ A_2_)] [(2 min(V(A_1_), V(A_2_))–V(A_1_ ∩ A_2_))]^-1^; [65]) and nestedness (*FD*_*nes*_ = [(|V(A1)—V(A2)| V(A1 ∩ A2)) ((V(A1) + V(A2)–V(A1 ∩ A2))] [((2 min(V(A1), V(A2))–V(A1∩ A2)]^-1^; [23, 70]). All functional β-diversity analyses were carried out using the mFD package in R 4.2.1 [68]. Just like in the taxonomic analysis, the values of these indexes ranged from 0 to 1. These calculations using the current, future-1 and future-2 matrixes allowed to determine the differences (Δ) from the historical values [70].

### Analysis

We carried out pairwise comparisons of the β-diversity value distributions obtained for the current, future-1, and future-2 compositional scenarios with that of the historical scenario using the Student’s *t*-test. These comparisons contrasted the taxonomic, as well as the functional values. The covariation of the changes in both dimensions of β-diversity was also examined using an analysis of correlation and regression of the differences with respect to the historical scenario, comparing the Δ*TD*_β_ *versus* Δ*FD*_β_; Δ*TD*_*tur*_ *versus* Δ*FD*_*tur*_ and Δ*TD*_*nes*_ *versus* Δ*FD*_*nes*_ values for the current, future-1, and future-2 scenarios. We calculated the Pearson’s correlation coefficient (*r*) for each of these comparisons, which was tested using Montecarlo randomization [71]. In this process, the *r* value was recalculated 1,000 times after the taxonomic diversity values were randomly reassigned [71]. The frequency distribution of the 1,000 pseudo-values obtained for *r* allowed to determine whether the observed value was a product of chance or not.

The slope (*m*) values obtained in the linear regression analyses between Δ*TD*_β_ and Δ*FD*_β_, Δ*TD*_*tur*_ and Δ*FD*_*tur*,_ and Δ*TD*_*nes*_ and Δ*FD*_*nes*_ were also tested using Montecarlo randomization [71]. The taxonomic index values (Δ*TD*_β_, Δ*TD*_*tur*_ and Δ*TD*_*nes*_) were redistributed 1,000 times and the slope (*m*) was recalculated each time, together with its functional counterpart. Thus, the randomized frequency distribution of slope (*m*) pseudo-values was obtained for each of the regressions in order to estimate the bilateral probability associated with the observed slope (*m*) value. Finally, we compared the slopes and the β-diversity indexes of the different compositional scenarios using the Student’s *t*-test once homoscedasticity was confirmed.

## Results

Under the historical scenario, mean values of taxonomic β-diversity, turnover, and nestedness were 0.608, 0.432, and 0.176, respectively (Table 1). Turnover (*TD*_*tur*_) was responsible for 70% of taxonomic β-diversity, and nestedness (*TD*_*nes*_) for 30%. Under the current scenario, the respective values were 0.584, 0.472, and 0.111 (Table 1), with a statistically significant drop in taxonomic β-diversity (by 3.9%; Table 1) and in nestedness (by 36.9%; Table 1), and a significant increase in turnover (by 9.3%; Table 1). Here, the turnover was responsible for 80% of β-taxonomic diversity, and nestedness for 20%. Similarly, lower β-diversity and nestedness values were obtained under the hypothetical future-1 and future-2 scenarios compared with the historical one (Table 1), while turnover increased to 0.443 under future-1 and to 0.467 future-2 scenarios (Table 1). The relative importance of turnover and nestedness in the two future scenarios remained at similar percentages as those under the current scenario (*i*.*e*., 80% and 20%, respectively).

**Table 1:** β-diversity distribution (X ± SD), considering its taxonomic (β-diversity, *TD*_β_; turnover, *TD*_*tur*_; nestedness, *TD*_*nes*_) and functional dimensions (β-diversity, *FD*_β_; turnover, *FD*_*tur*_; nestedness, *FD*_*nes*_) in 20 freshwater fish assemblages of continental Chile. The values were calculated for the Historical and Current compositional scenarios, as well as for two hypothetical scenarios (Future-1 and Future-2) that assume intensification of the extinction of native species. The statistical comparisons refer to contrasting all scenarios against the historical scenario (Wilcoxon’s signed-rank test).

	Taxonomic diversity								
	*TD*_β_			*TD*_*tur*_			*TD*_*nes*_		
	X ± SD	Z	*P*	X ± SD	Z	*P*	X ± SD	Z	*P*
Historical	0.608 ± 0.236			0.432 ± 0.296			0.176 ± 0.174		
Current	0.584 ± 0.217	4.9	<0.0001	0.472 ± 0.265	-3.7	<0.0001	0.111 ± 0.113	7.9	<0.0001
Future-1	0.542 ± 0.228	8.1	<0.0001	0.443 ± 0.262	0.7	0.465	0.099 ± 0.092	6.7	<0.0001
Future-2	0.569 ± 0.215	4.5	<0.0001	0.467 ± 0.271	-2	0.042	0.102 ± 0.11	6.2	<0.0001
	Functional diversity								
	*FD*_β_			*FD*_*tur*_			*FD*_*nes*_		
	X ± SD	Z	*P*	X ± SD	Z	*P*	X ± SD	Z	*P*
Historical	0.762 ± 0.33			0.317 ± 0.431			0.446 ± 0.402		
Current	0.53 ± 0.291	9.4	<0.0001	0.356 ± 0.343	-1.2	0.219	0.174 ± 0.162	7.4	<0.0001
Future-1	0.581 ± 0.296	8.1	<0.0001	0.335 ± 0.37	0.9	0.32	0.246 ± 0.233	6.1	<0.0001
Future-2	0.732 ± 0.229	4.1	<0.0001	0.427 ± 0.343	-3.6	<0.0001	0.305 ± 0.284	4.1	<0.0001

On the other hand, the functional dimension of β-diversity showed mean historical values of 0.762, 0.317, and 0.446 for β-diversity, turnover, and nestedness, respectively (Table 1). In this case, nestedness represented on average 60% of β-diversity values, while turnover represented 40%. Under the current scenario, mean β-diversity and nestedness values decreased to 0.530 (a reduction of 30.4%; Table 1) and to 0.174 (a reduction of 60.9%; Table 1), respectively. This reduction compared to the historical value was statistically significant in both cases (Table 1). The functional turnover increased to 0.356 (an increase of 12.3%; Table 1), which did not differ significantly from the historical value (Table 1). Interestingly, the percentage values of the importance of turnover and nestedness were reversed with respect to the historical values, being 67% and 33%, respectively. Under future-1 and future-2 scenarios, the relative importance of turnover and nestedness remained around 58% and 42%, respectively.

**Fig 2:**
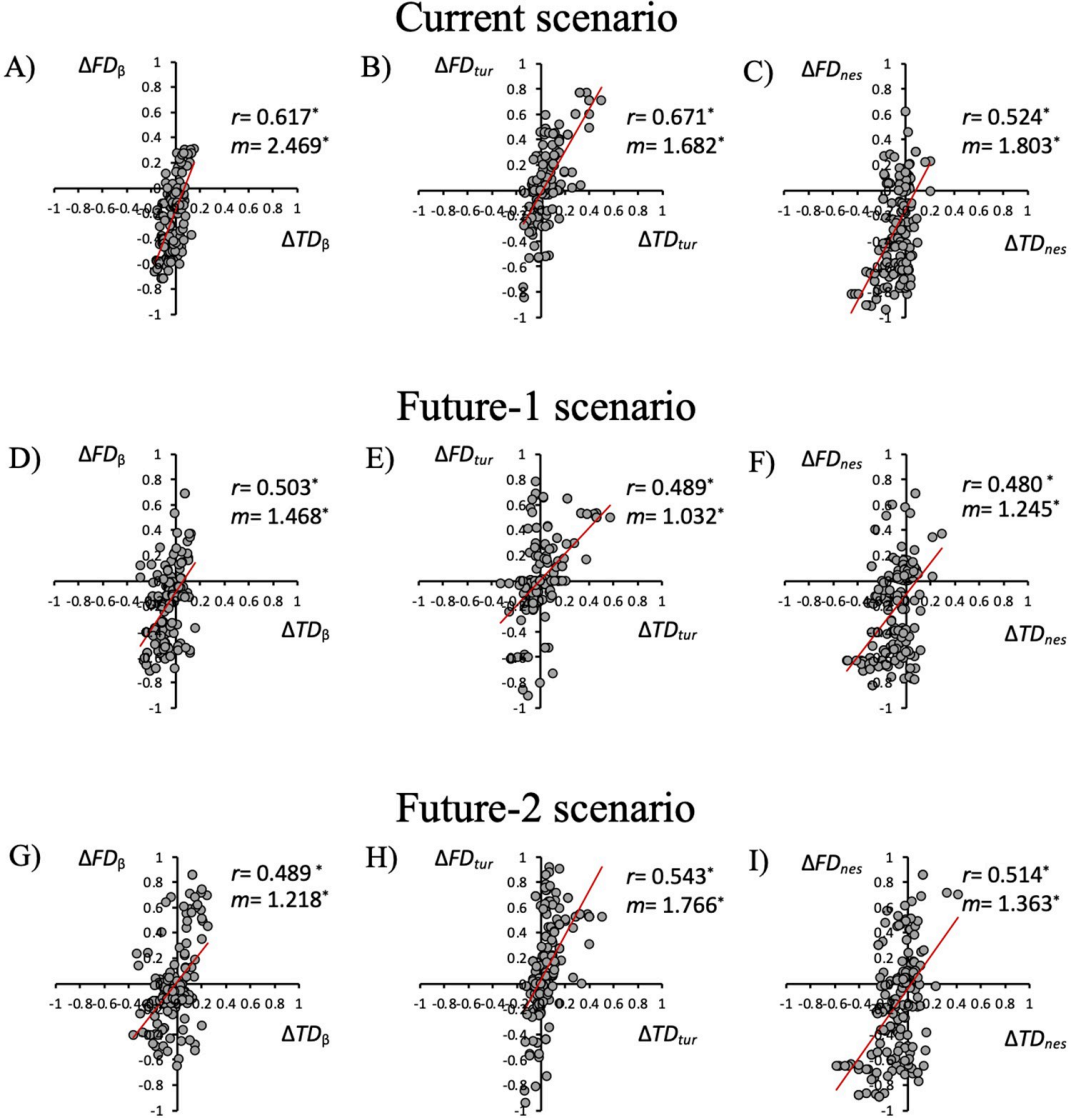
Regression/correlation between taxonomic (Δ*TD*_β_, Δ*TD*_*tur*_ and Δ*TD*_*nes*_) and functional (Δ*FD*_β_, Δ*FD*_*tur*_ and Δ*FD*_*nes*_) β-diversity, turnover, and nestedness for the current scenario and two hypothetical future scenarios (future-1 and future-2). 190 pairwise comparisons were used for each β-diversity index. The asterisk (*) indicates statistically significant correlation values (*r*) and/or regression slopes (*m*) that are statistically different from 1 (P < 0.05).

When compared, the taxonomic and functional differences (Δ) between the current and historical scenarios showed a non-random distribution and were positively and significantly correlated (Montecarlo randomizations; 0.671 > *r* > 0.524; P < 0.05; Fig 2A–2C). Regarding the regression slopes (*m*), these were all significant (Student’s *t* > 2.9; *P* < 0.05 in all cases) and indicate that the functional dimension was more affected by the compositional changes than its taxonomic counterpart (Fig 2A–2C). As for β-diversity, 67.4% of the comparisons showed a combined decrease in taxonomic and functional values (Δ*TD* < 0 and Δ*FD* < 0; Fig 2A), 21.1% showed an increase in taxonomic difference associated with a decrease in the functional dimension (Δ*TD* > 0 and Δ*FD* < 0; Fig 2A), and 11.1% showed an increase in taxonomic difference together with an increase in its functional counterpart (Δ*TD* > 0 and Δ*FD* > 0; Fig 2A). Concerning the current differences in turnover (see Fig 2B and 2C), the taxonomic as well as the functional facets showed a greater contrast density (36.8%) in the region of taxonomic and functional dimension increase (*i*.*e*., Δ*TD* > 0 and Δ*FD* > 0; Fig 2B), while 50.5% of nestedness contrast density was in the opposite region (*i*.*e*., Δ*TD* < 0 and Δ*FD* < 0; Fig 2C).

The contrasts between assemblage pairs were more scattered under future-1 and future-2 scenarios, as indicated by lower correlation and slope values (0.543 > *r* > 0.480; 1.766 > *m* > 1.032; see Fig 2D through 2I). Despite this, they show the same pattern as before: Taxonomic as well as functional β-diversity and nestedness were lower compared to the historical values, while turnover increased in both dimensions of diversity. In the case of β-diversity, the combined decline (Δ*TD*_β_ < 0 and Δ*FD*_β_ < 0) under the future-1 scenario occurred in 70.5% (Fig 2D), and in the case of nestedness (Δ*TD*_*nes*_ < 0 and Δ*FD*_*nes*_ < 0) in 47.9% of the contrasts (Fig 2F). Under the future-2 scenario, the combined decline of Δ*TD*_β_ and Δ*FD*_β_ occurred in 57.4% (Fig 2G), and in the case of nestedness (Δ*TD*_*nes*_ and Δ*FD*_*nes*_; Fig 2I) in 41.1% of the contrasts. Turnover showed a combined decline (Δ*TD*_*tur*_ < 0 and Δ*FD*_*tur*_ < 0) in 41.1% of the contrasts under the future-1 scenario (Fig 2E), while under the future-2 scenario a greater frequency of combined increase was observed (Δ*TD*_*tur*_ > 0 and Δ*FD*_*tur*_ > 0) in 42.6% of the contrasts (Fig 2H). Additionally, the taxonomic and functional β-diversity differences had significant slopes (*t* > 2.9; P < 0.05 in all of the cases) under both future scenarios, but their values were lower than those observed under the current scenario (*t* > 2.8; *P* > 0.05 in all of the cases), indicating that both dimensions changed in a similar way.

## Discussion

Our results show a joint reduction in taxonomic and functional β-diversity in the freshwater fish assemblages of continental Chile. This β-diversity loss was observed when the compositional scenario was compared between the historical and current scenarios. A similar tendency was shown under two hypothetical future scenarios, although with a lower magnitude. These results are consistent with previous studies in Chile, which found that taxonomic [42] and functional β-diversity [43] of fish assemblages have decreased over time, implying a process of biotic homogenization in both facets of diversity. Our current findings go beyond that, demonstrating that the taxonomic and functional facets of β-diversity change are positively correlated but with contrasting tendencies of their components of turnover and nestedness.

From a taxonomic point of view, the β-diversity loss was caused by the presence of 28 exotic species that have become naturalized in practically all hydrographic basins in Chile [39, 61]. The native diversity of Chilean freshwater fishes consists of 42 species [34], rendering exotics approximately one third of the freshwater ichthyofauna [34], with their presence doubling the species richness in several hydrographic basins [42]. Most of these exotic species come from North America, Europe, and Asia, and were introduced for fish farming [72] or as pest control agents [51, 61]. *Cyprinus carpio*, *Gambusia affinis*, *Gambusia holbrooki*, *Odontesthes bonariensis*, *Oncorhynchus mykiss*, and *Salmo trutta* are six of the most widely distributed exotic species in continental Chile, which are present in 14 of the 20 basins included in our study (S1 Table). In contrast, the extinction/extirpation of native species has played a minor role in causing homogenization, because only a single native species has become extinct to date (*Diplomystes chilensis*), which was originally restricted to a single basin ([34]; S1 Table). Additionally, the practice of introducing native species into new basins (*i*.*e*., extra-limital native *sensu* La Sorte *et al*. [73]) has not been documented in Chile [42, 74]. Thus, the introduction of exotic species, coupled with the virtual absence of extinction and relocation of native taxa sets the freshwater fish assemblages of continental Chile apart from those in other regions of the world–even within the Neotropics [75–79]–, with respect to the mechanisms that may cause taxonomic homogenization. Although the generalized response of the majority of the assemblages considered in this study was a reduction in taxonomic β-diversity (in 67.3% of the comparisons), said indicator increased in a smaller set (in 32.7% of the cases), basically due to the effect of the introduction of exotic species into only a few basins. In fact, around 50% of the exotic species were restricted to one or two basins (S1 Table), which increased taxonomic differentiation. Nevertheless, this process was overshadowed by the opposing trend, which had a greater quantitative effect on β-diversity.

In terms of the components of taxonomic β-diversity, we observed different responses in the values of turnover and nestedness in the assemblages studied when the historical and current ones were compared. While taxonomic turnover increased by 9.2% on average from the historical to the current value, nestedness decreased by 36.8% on average. These results indicate that while taxonomic turnover causes taxonomic differentiation of the assemblages [23], nestedness contributes to homogenization [23], overshadowing the previous effect.

From a functional point of view, the wide distribution of a small number of exotic species that possess common functional traits has contributed to the homogenization of the assemblages through the reduction of functional β-diversity over time. Such exotic species are *Ameiurus nebulosus*, *Cheirodon interruptus*, *Cnesterodon decemmaculatus*, *Cyprinus carpio*, *Gambusia affinis*, *G*. *holbrooki*, *Ictalurus punctatus*, and *Jenynsia multidentata*, which possess similar (or redundant) functional traits in comparison to native species, such as small body length (< 50 cm; except for *Cyprinus carpio* and *Ictalurus punctatus*), demersal habits, and generalist diet consisting mostly of invertebrates (S3 Table). Functional β-diversity loss was observed in 86.8% of the current-historical comparisons, while this loss was less marked in the taxonomic dimension. The partitioning of functional β-diversity into turnover and nestedness revealed counteracting responses in these components. While functional turnover increased from the historical scenario to the current one by 12.3%, nestedness showed an abrupt drop by 60.9% over the same period of time. Thus, while functional turnover caused functional β-diversity–and with it functional differentiation–of assemblages to increase, functional nestedness reduced it, causing functional homogenization instead [23].

Several studies have analyzed the covariation of taxonomic and functional facets in freshwater fish assemblages, demonstrating differing trends. For example, Campbell and Mandrak [29], Daga *et al*. [80], Dala-Corte *et al*. [81], and Kang *et al*. [82] found that taxonomic β-diversity loss (*i*.*e*., taxonomic homogenization) was associated with functional β-diversity gain (*i*.*e*., functional differentiation); that is, were negatively correlated. To the contrary, Su *et al*. [83], Toussaint *et al*. [84], and our study, showed that taxonomic β-diversity loss correlated positively with functional β-diversity loss; that is, causing simultaneous homogenization of both dimensions. These differences owe to a combination of factors [59, 85–87], which basically relate to how exotic and native species are distributed in space [11] and how invasion/extinction events affect taxonomic and functional structure [11, 15, 84]. In Chile, two (*Oncorhynchus mykiss* and *Salmo trutta*) of the six exotic species that contribute the most to taxonomic homogenization were present in all 20 basins studied (S1 Table). Nonetheless, because they both belong to the family Salmonidae, they intensified the response of the functional dimension while the other seven introduced salmonids (*Oncorhynchus gorbuscha*, *O*. *keta*, *O*. *kisutch*, *O*. *masou*, *O*. *nerka*, *O*. *tshawytscha*, and *Salmo salar*) show more localized distributions and more similar morpho-functional traits (S3 Table), a reflection of their close phylogenetic relations [17]. Therefore, this distribution pattern of exotics intensified functional rather than taxonomic homogenization [84].

Given that current regulations restrict the introduction of new species to Chile and the translocation of those that are already present [50], this factor was not considered when constructing future scenarios. Thus, future-1 and future-2 were used to predict the tendency of the taxonomic and functional dimensions of β-diversity as a response to only extinction events. In general, extinction events that involved either Endangered or Vulnerable species showed that the trend of β-diversity loss remained constant. The future-1 scenario simulated the extinction of all “Endangered” species (n = 14), several of which are only present in one or a few assemblages (S1 Table). More precisely, each Endangered fish species was distributed across four basins on average, the mode being two basins per species. Therefore, the disappearance of these Endangered taxa means the disappearance of taxonomic entities that are unique to each assemblage, then causing taxonomic homogenization [42]. The future-2 scenario simulated the extinction of both Endangered and Vulnerable species, thus adding 11 new possible extinction events (n_total_ = 25). The vulnerable *Basilichthys microlepidotus*, *Galaxias maculatus*, *Odonthestes brevianalis*, and *Trichomycterus areolatus* were among the native fishes with the widest distribution (S1 Table). The distribution of “Vulnerable” fishes was 11 basins on average, the mode being eight basins per species. Thus, our results showed that this set of Vulnerable species has a wider distribution on average, and their disappearance would thus entail the loss of taxonomic entities that are shared between assemblages, causing homogenization that is less intense than that resulting from the loss of only Endangered species [42].

From a functional viewpoint, the trend above was reversed. Examining how functional β-diversity was affected, we observed a trend of reduced intensity in functional homogenization and a higher proportion of comparisons that represent differentiation from the historical scenario (current scenario: Δ*FD*_β_ = 12.6%; future-1 scenario: Δ*FD*_β_ = 17.9%; future-2 scenario: Δ*FD*_β_ = 28.9%). This was because the functional diversity of native fish species derives from a set of taxa with specialized traits that differ from those exhibited by the exotic ichthyofauna. Then, the extinction of native species would cause the disappearance of unique functional traits [88].

The state of conservation of Chilean freshwater fishes is critical, with 88% of the species presenting conservation issues [42]. Therefore, the extinction of these fishes is considered highly likely in the medium term, unless effective measures of protection are implemented [48]. With this in mind, our results shed light on how two components of this diversity (taxonomic and functional) have been affected historically and how they are expected to change in the future as a result of the introduction of exotic species and the possible extinction of native ones. First of all, it is necessary to improve our knowledge concerning the less studied aspects of biodiversity: Their phylogenetic and evolutionary dimensions [17]. In fact, these components of diversity have not yet been analyzed in Chilean assemblages [42], despite their link with the provision of ecosystem services [89–91]. In qualitative terms, it has been established that the Chilean ichthyofauna, given its biogeographic isolation, harbors a relictual taxonomic diversity [36]. This is especially true in the case of several catfish species from the Diplomystidae, Nematogenyidae, and Trichomycteridae (Siluriformes), which are all representatives of a Tertiary ichthyofauna [36], characterized by traits that are considered plesiomorphic [92]. Therefore, the current presence of exotic species from the Northern Hemisphere that belong to lineages that were absent in this region, and the likely extinction of native species from relictual lineages, highlight already severe alterations in phylogenetic structure and diversity.

In summary, our study shows that the freshwater ichthyofauna of continental Chile is undergoing biotic homogenization, as evidenced by β-diversity loss. This process involves the facets of taxonomic and functional β-diversity, which are positively correlated between the historical and current compositions and have decreased by 3.9% and 30.4%, respectively. Additionally, both of these dimensions show that the homogenization process is caused by nestedness, and while turnover contributes to differentiation (taxonomic and functional), its importance is overshadowed by nestedness. Taxonomic homogenization is caused by a small number of exotic species (six out of nine Salmonids among a total of 28 exotic fishes) that are widely distributed in Chilean basins and share a set of functional traits with those that have a more restricted distribution. This scenario causes a more intense functional homogenization than that in taxonomic terms. Our projections, assuming an intensification of the extinction of Endangered and Vulnerable native species, indicate that further homogenization will continue, though at lower rate. Progress in conservation efforts and the implementation of specific protective measures are paramount, considering the unique characteristics of the freshwater fish assemblages of continental Chile.

## Supporting information

S1 Table Data matrix with freshwater fish species, origin, status, and occurrence at 20 basins in continental Chile. For each basin the occurrence of native and exotic species is indicated by 1 = Present; 0 = Absent; 1/0 = Extirpated. The conservation status of native species is indicated as: CR = Critically endangered; EN = Endangered; VU = Vulnerable; NT = Near threatened; LC = Least concern;— = Not evaluated (MMA 2021). For native and exotic species, brackets are ordered as: [historical presence, current presence, presence in Future 1 assemblage], presence in Future 2 assemblage].
(XLSX)

S2 Table Functional traits used to describe fish functional diversity. Native and exotic fish traits and functional roles from Rojas *et al*. (2020) (https://doi.org/10.1016/j.gecco.2020.e01355).
(XLSX)

S3 Table Native and exotic fish traits and functional roles. (XLSX)
